# Recognition of Brain Metastases Using Gadolinium-Enhanced SWI MRI: Proof-of-Concept Study

**DOI:** 10.3389/fneur.2020.00005

**Published:** 2020-02-11

**Authors:** Joel Ceballos-Ceballos, Diego A. Loza-Gallardo, Marco A. Barajas-Romero, Carlos Cantú-Brito, Sergio Iván Valdés-Ferrer

**Affiliations:** ^1^Division of Neuroradiology, Department of Radiology, Hospital San Javier, Guadalajara, Mexico; ^2^Department of Surgical Neurology, Hospital San Javier, Guadalajara, Mexico; ^3^Department of Neurology, Instituto Nacional de Ciencias Médicas y Nutrición Salvador Zubirán, Mexico City, Mexico; ^4^Department of Infectious Diseases, Instituto Nacional de Ciencias Médicas y Nutrición Salvador Zubirán, Mexico City, Mexico; ^5^Center for Biomedical Science, Feinstein Institute for Medical Research, Manhasset, NY, United States

**Keywords:** brain metastasis, MRI, SWI, susceptibility-weighted image, gadolinium, metastasis diagnosis

## Abstract

**Background and purpose:** SWI MRI, a T2^*^-dominant MRI sequence with T1 *shine-through* effect, uses intrinsic structural susceptibility to create enhancement among brain structures. We evaluated whether gadolinium-enhanced SWI (SWI-Gd) improves brain metastasis detection in combination with other MRI sequences.

**Materials and methods:** MRI images of 24 patients (46 studies) were prospectively acquired using a 1.5-T scanner. T1-weighted, unenhanced SWI (SWI-U) and SWI-Gd were evaluated blindly to clinical features by two board-certified radiologists.

**Results:** SWI-Gd revealed more significant metastatic lesions than either T1-Gd or SWI-U (*p* = 0.0004 for either comparator sequence). Moreover, SWI-Gd revealed more lesions only for those patients with ≤5 lesions on T1-Gd (*n* = 30 studies from 16 patients; *p* = 0.046). Performing SWI-Gd added <5 min of scanning time with no further additional risk.

**Conclusions:** Our findings suggest that, when added to T1-Gd and other common sequences, SWI-Gd may improve the diagnostic yield of brain metastases with only a few extra minutes of scanning time and no further risk than that of a regular gadolinium-enhanced MRI.

## Introduction

Cerebral metastases are the most common form of brain tumors in adults (1, 2), are a significant source of morbidity and mortality, and have direct implications on the treatment and prognosis of the primary tumor (3, 4).

Contrast-enhanced magnetic resonance imaging (MRI) is the standard for diagnosing brain metastases (5); however, there is no one-size-fits-all MRI protocol for their evaluation (6). Treatment of brain metastases depends on a number of tumor and host factors, as well as lesion location and surgical accessibility, for which an adequate MRI staging is crucial (5). Taking advantage of already established MRI methods–with simple modifications–can potentially result in earlier detection of metastatic lesions.

Susceptibility-weighted imaging (SWI) is an MRI technique that takes advantage of intrinsic magnetic susceptibility differences between adjacent tissues (7), leading to a better distinction of inter- and intratissue characteristics (8, 9). SWI is routinely performed without the use of gadolinium contrast enhancement and is used clinically to evaluate a number of neurological conditions (10, 11).

Although SWI is a T2^*^-dominant sequence, there is also a so-called *T1 shine-through* effect, in which lesions preserve on SWI the characteristics expected on T1-weighted images (12). Hence, gadolinium can hypothetically enhance lesions on SWI and, by creating further intratissue contrast, unmask small lesions early in the course of metastatic seeding.

Gadolinium enhancement has been used experimentally in SWI for evaluating primary gliomas, where it has shown promising results on staging, grading, and even determining the aggressiveness of primary brain tumors (13). SWI-Gd has been recently shown to be helpful for the detection of blood–brain barrier dysfunction in patients with multiple sclerosis (14), suggesting that it can also improve the imaging assessment of brain metastases. Hence, gadolinium enhancement in SWI (SWI-Gd) can potentially reveal certain characteristics of cerebral metastases not observed otherwise. The present study was therefore designed to investigate if SWI-Gd can improve identifying metastatic brain lesions in comparison to the usual (T1-Gd and SWI-U) MRI sequences.

## Methods

### Ethics

The study was approved by the Institutional Ethics Review Board of Hospital San Javier, Guadalajara, Mexico. All patients signed the informed consent.

### Study Design and Selection of Participants

This study was designed as a prospective one. All participants were adults (≥18 years old) with a histologically confirmed systemic tumor who came for MRI evaluation either in search of cerebral metastases or to evaluate previously determined metastatic disease. Demographic data, as well as specific details about the diagnosis of the primary and metastatic tumor, and other diagnostic data were acquired retrospectively from hospital records.

### MRI Protocol

MRI studies were acquired on a 1.5-T scanner (Achieva; Philips Healthcare, Best, The Netherlands) using a 16-channel coil. After signing the informed consent, participants underwent a standard unenhanced MRI protocol [T1-, T2-, diffusion-weighted, and fluid-attenuated inversion recovery (FLAIR)], followed by T1-GD images (duration of acquisition: 3'17”) and SWI-Gd (duration of acquisition: 3'26”), instead of only acquiring the routine T1-Gd images. The order of sequence acquisition postcontrast infusion was as follows: (1) Perfusion-weighted (duration: 1'11”); (2) T1-weighted (duration: 3'17”); SWI (duration: 3'26”). Therefore, gadolinium-enhanced T1-weighted images were acquired 1'11” post-infusion, while SWI-Gd were acquired 4'38” post-infusion. Lesions of interest were defined *a priori* as follows: in SWI-U, as single or multiple, hypointense or hyperintense lesions, as previously reported (15). In gadolinium-enhanced sequences, lesions show nodular or ring enhancement. MRI studies were performed from September 2017 to August 2018. Studies that had artifacts interfering with the interpretation or those suggestive of an alternative diagnosis were excluded from the study.

Contrast-enhanced with fast field echo (FFE) T1-weighted images were acquired using the following parameters: slice thickness 0.55 mm; FOV 230 × 183 × 142 mm^3^; matrix 256 × 159; TR/TE/TI 11,000/130/2,800 ms; and acquisition time 5 min and 8 s. SWI images were acquired with a flow-compensated 3D gradient-echo method using the following parameters: FOV, 230 × 187 × 130 mm^3^; matrix, 244 × 186 mm^3^; voxel size, 1.0 × 1.0 × 2.0 mm^3^; voxel volume, 2 mm^3^; TR/TE, 51/60 ms; slice thickness, 1 mm; flip angle = 20°; acquisition time, 3 min and 26 s. For image reconstruction on SWI, we used raw data, as well as minimum intensity projection (MinIP). Gadolinium-enhanced SWI and T1-weighted images were repeated after intravenous administration of 0.2 ml/kg (0.1 mmol/kg) of gadoterate meglumine (Dotarem® Guerbet; Paris, France), which was infused as a bolus at a rate of 2.0 ml/s. The order of imaging acquisition was consistent between participants. SWI images were reviewed simultaneously in phase and magnitude; phase was used to detect the paramagnetic/diamagnetic signal that is suggestive of blood or calcium content. All images were analyzed blindly to patient information by two radiologists; one of them (JC-C) is a neuroradiologist with 30 years of diagnostic experience, while the second one (DAL-G) has 3 years of neuroimaging diagnostic experience. Images were analyzed jointly by both reviewers, and individual lesions, as well as lesion burden, were determined by consensus.

### Statistical Analysis

Statistical analysis was performed using Prism version 6 (GraphPad Software, San Diego, CA). Images were analyzed independently by two observers, and the number of metastatic lesions was counted manually on unenhanced SWI and T1-weighted followed by Gd-enhanced sequences. Continuous data such as means and standard deviations were obtained. *Student t* and one-way ANOVA tests were used to compare differences between groups, and *p* < 0.05 were considered statistically significant.

## Results

### Sample

Thirty-five patients agreed to participate; however, six did not sign the informed consent and were therefore excluded from the study; of the 29 remaining patients, five patients had to be excluded due to technical reasons. Our final sample included 24 patients (19 female, 5 male), with a total of 58 MRI studies, of which 12 studies were excluded from the analysis due to movement artifacts, leaving us with 46 MRI studies that were considered of good quality for analysis (on average, 1.9 studies/patient).

### Demographic and Tumor-Specific Baseline Characteristics

Patients presented with a wide variety of clinical manifestations, but the presence of headache, altered mental status, and focal neurological signs were the most commonly recorded (Table 1).

**Table 1:** Clinical manifestations of brain metastases (patients had in general more than one clinical manifestation).

**Tumor (*N* = 24)**	**Frequency (%)**
Asymptomatic/incidental (*n* = 3)	12.5
Headache (*n* = 10)	41.6
Altered mental status (*n* = 5)	20.8
Focal signs (*n* = 4)	16.7
Seizures (*n* = 3)
• Status epilepticus (*n* = 1)	12.5
Cerebellar/ataxia: (*n* = 3)	12.5
Cranial nerve signs (*n* = 2)	8.3
Pain/sensory loss (*n* = 2)	8.3
Vertigo/dizziness (*n* = 2)	8.3
Visual disturbances (*n* = 2)	8.3
Gait disorders (*n* = 2)	8.3
Intracranial hypertension (*n* = 2)	8.3

The mean age at diagnosis of the primary tumor was 56.4 ± 16.1 years, while the age at diagnosis of metastatic disease was 57.8 ± 16.5 years. The mean time from diagnosis of the primary tumor to metastatic brain disease was 372 ± 519 days. The most common tumor was breast cancer (*n* = 11; 45.8%) followed by lung (*n* = 7; 29.5%), and colon (*n* = 2; 8.3%) cancer; there was one patient each with pancreatic, kidney, melanoma, or hematopoietic cancer.

### MRI Findings

#### Burden of Metastatic Brain Disease

**Figure 1:**
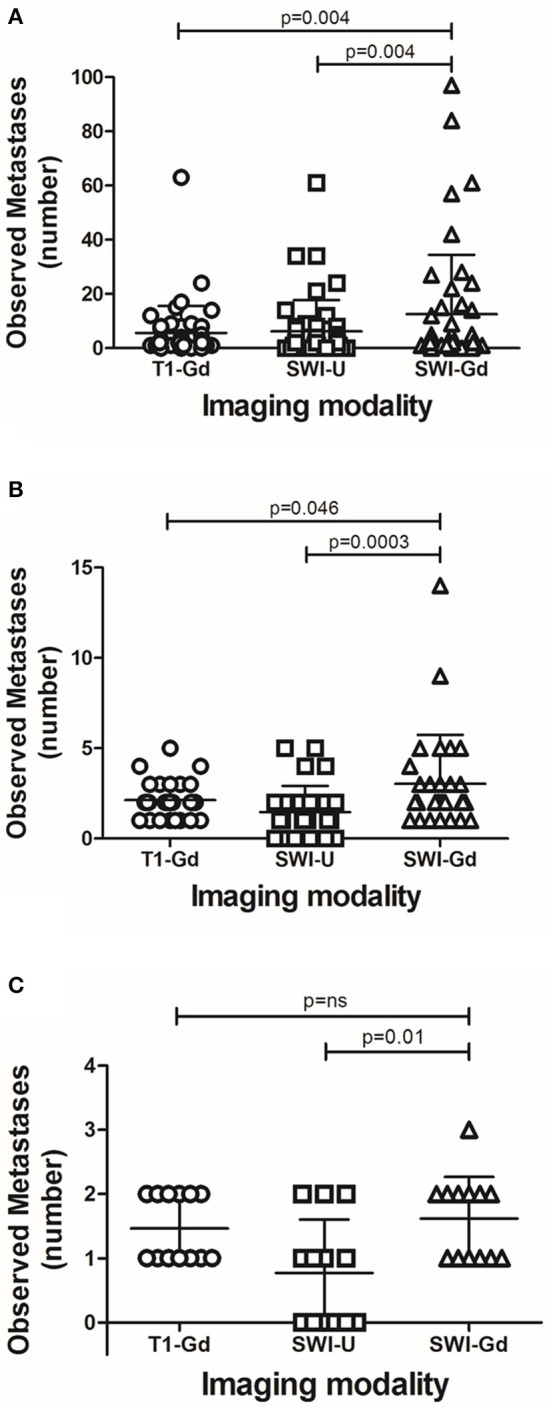
Detection of metastatic brain lesions by different MRI sequences. The number of detected metastases by each of the analyzed MRI sequences with all cases **(A)**; those with five or fewer lesions **(B)**; or those with two or fewer lesions **(C)**. Each dot represents a patient. Error bars represent median ± interquartile range. Statistical values represent differences between groups, and a value of *p* ≤ 0.05 was considered statistically significant.

In T1-Gd, we observed an average of 5.61 ± 10.02 brain metastases; in SWI-U, we observed an average of 6.22 ± 11.61 brain metastases. In contrast, in the SWI-Gd, we observed, on average, 12.52 ± 21.95 brain metastases. SWI-Gd was statistically superior to either reference sequence (one-way ANOVA, *p* = 0.0009; intergroup Student's *t*-test, *p* = 0.0004) (Figure 1A).

A sub-analysis of MRI studies independently of the burden of metastasis showed that, in 24 out of 46 sets of images obtained from 14 patients, SWI-Gd revealed one or more enhancing lesions that had not been observed in T1-Gd; in comparison, T1-Gd revealed more enhancing lesions than those seen by SWI-Gd in only one case. We observed no difference in the number of metastatic lesions in the remaining studies.

**Figure 2:**
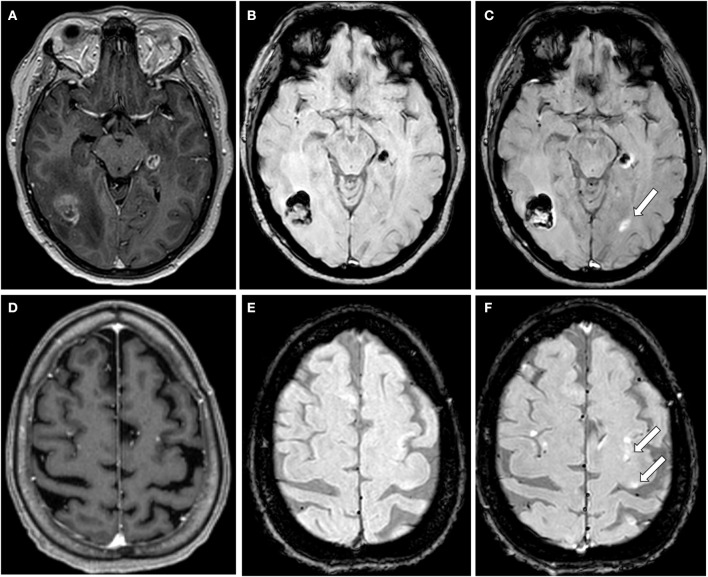
Illustrative cases of lesions seen only with SWI-Gd. **(A–C)** MRI from a 45 years old female patient diagnosed with breast cancer and preexisting metastases on the right parieto-occipital region and a second one on the fifth left temporal gyrus. T1-Gd shows heterogeneous enhancement on both lesions **(A)** that were heterogeneous but predominantly hypointense on SWI-U **(B)**. SWI-Gd showed annular enhancement and edema of the previously described lesions, as well as an otherwise not seen lesion (arrow) in the left occipital lobe with annular enhancement **(C)**. **(D–F)** MRI from a 58 years old female with pancreatic cancer. T1-Gd shows three small enhancing lesions: two in the right frontal lobe; another in the left frontal lobe **(D)**, that were not observed with SWI-U **(E)**. SWI-Gd shows the lesions observed in T1-Gd, as well as two previously unseen lesions (arrows) **(F)**.

We then wondered if SWI-Gd would result in an improved ability to determine the number of brain lesions in patients with a smaller burden of metastatic disease (Figure 2). First, we reviewed MRI scans from patients with ≤5 brain metastases in T1-Gd. A total of 30 MRIs from 16 patients were analyzed. In this group, T1-Gd revealed a mean of 2.13 ± 1.0 metastases; SWI-U, 1.47 ± 1.46 metastases; while SWI-Gd, 3.03 ± 2.71 metastases. Here, we also observed that SWI-Gd was statistically superior (one-way ANOVA, *p* = 0.003; intergroup Student's *t*-test, *p* = 0.046 vs. T1-Gd and *p* = 0.0003 vs. SWI-U) (Figure 1B).

Finally, we reviewed MRI scans from patients with ≤2 brain metastases in T1-Gd. A total of 20 MRIs from 11 patients were analyzed. In this group, T1-Gd revealed 1.6 ± 0.5 metastases; SWI-U, 1.0 ± 0.86 metastases; and SWI-Gd, 1.85 ± 0.93 metastases. At this level (two or fewer lesions or visible on T1-Gd), SWI-Gd was superior to SWI-U (Student's *t*-test, *p* = 0.01), but not statistically different to T1-Gd (Figure 1C).

#### Non-oncologic Findings

Images suggestive of microbleeds were observed in three scans using SWI-U and four scans using SWI-Gd (*p* = ns). Microbleeds were concurrent on SWI-U and SWI-Gd in two patients with breast cancer metastases and one patient with hematopoietic cancer metastases. The differing case occurred in one patient with malignant melanoma metastases. Six patients had non-specific white matter intensities suggestive of microangiopathy; three had dilated perivascular spaces; two patients had intrasellar arachnoidocele; and one had leukoaraiosis associated with radiotherapy. As expected, non-oncological findings were consistent between studies.

## Discussion

Here, we present compelling preliminary evidence suggesting that SWI-Gd MRI increases the ability to recognize brain metastases from extracerebral tumors (Figure 2).

Ever since its inception in the clinical arena, brain MRI changed the way intracranial metastases are diagnosed (16). Among the common MRI sequences available, SWI takes advantage of intrinsic tissue magnetic characteristics to generate intrinsic contrast. SWI generates invaluable information about cerebral vasculature, deoxyhemoglobin in veins, hemorrhage and microbleeds, iron, and calcium deposition, and neovascularization, all without the need for contrast agents (17–19). SWI is therefore used routinely to evaluate a number of neurological conditions, including diffuse axonal injury, stroke, multiple sclerosis, or cerebral amyloid angiopathy, to name a few (10, 11). Recent evidence suggests that gadolinium can be useful in susceptibility-weighted MRI (14) hypothetically by taking advantage of the so-called *T1 shine-through* effect (12), potentially leading to improved detection of brain metastases, lesions know to be small, complex, and heterogeneous.

The diagnostic sensitivity of different MRI sequences (including T1-Gd and SWI-U) for the detection of brain metastases has been addressed before. In the case of brain metastases from systemic melanoma or breast cancer, T1-Gd has been shown to be better than other modalities (15, 20). Moreover, in direct comparison with T1-Gd for detecting melanoma metastases, SWI-U has shown no diagnostic advantage (21). To our knowledge, the present study is the first one to prospectively evaluate the added value of SWI-Gd for the potential detection of brain metastases. Here, we observed that SWI-Gd reveals more lesions with a greater level of detail than the usual sequences. This held true across the whole sample, as well as when we selected those participants with ≤5 lesions. However, we failed to observe differences when only those with ≤2 were considered, probably reflecting that SWI-Gd has no role at the lower burden of metastatic disease, but may also reflect a lack of power to detect differences due to the small sample size. Interestingly, some lesions that were visible in SWI-U became clearer after contrast administration; however, others were only observable only in SWI-Gd, even with a side-by-side unblinded comparison with SWI-U or T1-Gd. As expected, we did not observe improved detection of hemorrhagic lesions between SWI-U and SWI-Gd. This may be also due to the small sample size, as we observed microhemorrhages on a very small subset (three patients on SWI-U; four patients on SWI-Gd).

SWI-Gd has been evaluated for primary brain tumors with mixed results. In gliomas, SWI is useful in determining certain characteristics of the tumor, including growth potential (low- and high-grade) (22), intratumor vasculature (23, 24), and treatment response (25, 26). To our knowledge, our study is the first to analyze SWI-Gd for metastatic brain disease; its utility in the diagnosis of primary gliomas suggests that it can be a useful addition to the standard MRI protocol for evaluating brain metastases.

In the present study, the interval between gadolinium infusion and SWI acquisition was around 5 min. The timing between infusion of gadolinium and MRI acquisition can critically interfere with image enhancement, where delayed postcontrast acquisition (~20 min) may theoretically result in improved rates of lesion detection (27). However, shorter intervals (as in the present study, in which the delay was 4'38”) have shown minimal, if any, impact on image quality or detection ability (28).

Due to the non-invasive nature of the present study, while all primary tumors were histologically confirmed, we did no obtain histological confirmation from the observed metastatic lesions. However, preclinical evidence derived from animal models of tumor metastases suggests that gadolinium-enhancing lesions correspond to metastatic seeding (29).

Our study has several limitations. First of all, we have a relatively small sample size due to the exploratory (proof-of-concept) design; therefore, our findings will have to be replicated or rejected in future studies. Other important limitations include a mixture of primary tumors, analysis of parenchymal (but not leptomeningeal) lesions, as well as expected differences in tumor biology among participants. Also, we did not perform a concordance analysis between MRI evaluators. While some patients were scanned only once, some others were scanned as many as four times during the duration of the present study, and we did not perform an intrasubject analysis. Those limitations, inherent to a proof-of-concept study, will, therefore, need to be validated in larger, tumor-specific, cohorts.

The study has also some technical limitations. In every case, T1-Gd was acquired before SWI-Gd; while the span between both was short, it is possible that the observed advantage of SWI-Gd was due in part to delayed enhancement. We plan to evaluate this in a follow-up study. Also, we used a 1.5-T scanner, something that can be seen as a limitation as well as an advantage: a limitation as it is known that more powerful scanners have better sensibility and require half the dose of gadolinium to achieve similar results; on the opposite end, 1.5-T scanners are widely available, making the results more easily generalizable.

In conclusion, here we observe for the first time that SWI-Gd can be a valuable addition to the detection of brain metastases. By taking advantage of gadolinium contrast, SWI-Gd may improve the detection of brain metastases when added to the standard contrast-enhanced MRI sequences without further risk (as the patient is already receiving gadolinium) and with only a few extra minutes of scanning.

## Data Availability Statement

All datasets generated for this study are included in the article/supplementary material.

## Ethics Statement

The studies involving human participants were reviewed and approved by Institutional Ethics Review Board of Hospital San Javier, Guadalajara, Mexico. The patients/participants provided their written informed consent to participate in this study.

## Author Contributions

JC-C, DL-G, and SV-F conceived the study. Data was collected by JC-C, DL-G, and MB-R. Data were analyzed by SV-F and JC-C. The manuscript was written by SV-F, CC-B, and JC-C. All authors reviewed and approved the final version.

### Conflict of Interest

The authors declare that the research was conducted in the absence of any commercial or financial relationships that could be construed as a potential conflict of interest.

## References

[1] Nayak L Lee EQ Wen PY. Epidemiology of brain metastases. Curr Oncol Rep. (2012) 14:48–54. 10.1007/s11912-011-0203-y 22012633

[2] Barnholtz-Sloan JS Sloan AE Davis FG Vigneau FD Lai P Sawaya RE. Incidence proportions of brain metastases in patients diagnosed (1973 to 2001) in the Metropolitan Detroit Cancer Surveillance System. J Clin Oncol. (2004) 22:2865–72. 10.1200/JCO.2004.12.149 15254054

[3] Mcneill KA. Epidemiology of brain tumors. Neurol Clin NA. (2016) 34:981–98. 10.1016/j.ncl.2016.06.014 27720005

[4] Sankowski R Mader S Valdés-Ferrer SI. Systemic inflammation and the brain: novel roles of genetic, molecular, and environmental cues as drivers of neurodegeneration. Front Cell Neurosci. (2015) 9:28. 10.3389/fncel.2015.00028 25698933 PMC4313590

[5] Soffietti R Abacioglu U Baumert B Combs SE Kinhult S Kros JM. Diagnosis and treatment of brain metastases from solid tumors: guidelines from the European Association of neuro-oncology (EANO). Neuro Oncol. (2017) 19:162–74. 10.1093/neuonc/now241 28391295 PMC5620494

[6] Zoccatelli G Alessandrini F Beltramello A Talacchi A Advanced magnetic resonance imaging techniques in brain tumours surgical planning. J Biomed Sci Eng. (2013) 06:403–17. 10.4236/jbise.2013.63A051

[7] Reichenbach JR Venkatesan R Schillinger DJ Kido DK Haacke EM. Small vessels in the human brain: MR venography with deoxyhemoglobin as an intrinsic contrast agent. Radiology. (2014) 204:272–7. 10.1148/radiology.204.1.9205259 9205259

[8] Haacke EM Xu Y Cheng Y-CN Reichenbach JR. Susceptibility Weighted Imaging (SWI). Magn Reson Med. (2004) 52:612–8. 10.1002/mrm.20198 15334582

[9] Haacke E Mittal S Wu Z Neelavalli J Cheng Y-C. Susceptibility-weighted imaging: technical aspects and clinical applications, part 1. Am J Neuroradiol. (2009) 30:19–30. 10.3174/ajnr.A1400 19039041 PMC3805391

[10] Mittal S Wu Z Neelavalli J Haacke EM. Susceptibility-weighted imaging: technical aspects and clinical applications, part 2. Am J Neuroradiol. (2009) 30:232–52. 10.3174/ajnr.A1461 19131406 PMC3805373

[11] Santhosh K Kesavadas C Thomas B Gupta AK Thamburaj K Kapilamoorthy TR. Susceptibility weighted imaging: a new tool in magnetic resonance imaging of stroke. Clin Radiol. (2009) 64:74–83. 10.1016/j.crad.2008.04.022 19070701

[12] Hsu CCT Haacke EM Heyn CC Watkins TW Krings T. The T1 shine through effect on susceptibility weighted imaging: an under recognized phenomenon. Neuroradiology. (2018) 60:235–7. 10.1007/s00234-018-1977-5 29330657

[13] Trattnig S Pinker K Ba-Ssalamah A Nöbauer-Huhmann IM. The optimal use of contrast agents at high field MRI. Eur Radiol. (2006) 16:1280–7. 10.1007/s00330-006-0154-0 16508769

[14] Do Amaral LLF Fragoso DC Nunes RH Littig IA Da Rocha AJ. Gadolinium-enhanced susceptibility-weighted imaging in multiple sclerosis: optimizing the recognition of active plaques for different MR imaging sequences. Am J Neuroradiol. (2019) 40:614–9. 10.3174/ajnr.A5997 30846435 PMC7048498

[15] Deike-Hofmann K Thünemann D Breckwoldt MO Schwarz D Radbruch A Enk A. Sensitivity of different MRI sequences in the early detection of melanoma brain metastases. PLoS ONE. (2018) 13:e0193946. 10.1371/journal.pone.0193946 29596475 PMC5875773

[16] Fink K Fink J. Imaging of brain metastases. Surg Neurol Int. (2013) 4:S209–19. 10.4103/2152-7806.111298 23717792 PMC3656556

[17] Liu S Buch S Chen Y Choi HS Dai Y Habib C. Susceptibility-weighted imaging: current status and future directions. NMR Biomed. (2017) 30:e3552. 10.1002/nbm.3552 27192086 PMC5116013

[18] Boeckh-Behrens T Lutz J Lummel N Burke M Wesemann T Schöpf V. Susceptibility-weighted angiography (SWAN) of cerebral veins and arteries compared to TOF-MRA. Eur J Radiol. (2012) 81:1238–45. 10.1016/j.ejrad.2011.02.057 21466929

[19] Shams S Martola J Cavallin L Granberg T Shams M Aspelin P. SWI or T2*: which MRI sequence to use in the detection of cerebral microbleeds? The Karolinska Imaging Dementia Study. Am J Neuroradiol. (2015) 36:1089–95. 10.3174/ajnr.A4248 25698623 PMC8013035

[20] Franceschi AM Moschos SJ Anders CK Glaubiger S Collichio FA Lee CB. Use of Susceptibility-Weighted Imaging (SWI) in the detection of brain hemorrhagic metastases from breast cancer and melanoma. J Comput Assist Tomogr. (2016) 40:803–5. 10.1097/RCT.0000000000000420 27636126 PMC5027959

[21] Schwarz D Niederle T Münch P Hielscher T Hassel JC Schlemmer H. Susceptibility-weighted imaging in malignant melanoma brain metastasis. J Magn Reson Imaging. (2019) 50:1251–9. 10.1002/jmri.26692 30793419

[22] Warmuth C Günther M Zimmer C. Quantification of blood flow in brain tumors: comparison of arterial spin labeling and dynamic susceptibility-weighted Contrast-enhanced MR imaging. Radiology. (2003) 228:523–32. 10.1148/radiol.2282020409 12819338

[23] Li C Ai B Li Y Qi H Wu L. Susceptibility-weighted imaging in grading brain astrocytomas. Eur J Radiol. (2010) 75:e81–5. 10.1016/j.ejrad.2009.08.003 19726149

[24] Di Ieva A Göd S Grabner G Grizzi F Sherif C Matula C. Three-dimensional susceptibility-weighted imaging at 7 T using fractal-based quantitative analysis to grade gliomas. Neuroradiology. (2013) 55:35–40. 10.1007/s00234-012-1081-1 22903580

[25] Law M Oh S Babb JS Wang E Inglese M Zagzag D Low-grade gliomas: dynamic susceptibility-weighted contrast-enhanced perfusion MR imaging—prediction of patient clinical purpose. Radiology. (2006) 238:658–67. 10.1148/radiol.2382042180 16396838

[26] Hori M Mori H Aoki S Abe O Masumoto T Kunimatsu S. Three-dimensional susceptibility-weighted imaging at 3 T using various image analysis methods in the estimation of grading intracranial gliomas. Magn Reson Imaging. (2010) 28:594–8. 10.1016/j.mri.2010.01.002 20233645

[27] Essig M Anzalone N Combs SE Dörfler A Lee SK Picozzi P. MR imaging of neoplastic central nervous system lesions: review and recommendations for current practice. Am J Neuroradiol. (2012) 33:803–17. 10.3174/ajnr.A2640 22016411 PMC7968800

[28] Cohen-Inbar O Xu Z Dodson B Rizvi T Durst CR Mukherjee S. Time-delayed contrast-enhanced MRI improves detection of brain metastases: a prospective validation of diagnostic yield. J Neurooncol. (2016) 130:485–94. 10.1007/s11060-016-2242-6 27568036

[29] Heyn C Ronald JA Ramadan SS Snir JA Barry AM MacKenzie LT. *In vivo* MRI of cancer cell fate at the single-cell level in a mouse model of breast cancer metastasis to the brain. Magn Reson Med. (2006) 56:1001–10. 10.1002/mrm.21029 17029229

